# Fructose 1,6-*bis*phosphatase: getting the message across

**DOI:** 10.1042/BSR20190124

**Published:** 2019-03-06

**Authors:** David J. Timson

**Affiliations:** School of Pharmacy and Biomolecular Sciences, University of Brighton, Huxley Building, Lewes Road, Brighton BN2 4GJ, U.K.

**Keywords:** allosteric enzyme, FBPase, fructose 1,6-bisphosphatase deficiency, gluconeogenesis, renal cancer, type II diabetes

## Abstract

Fructose 1,6-*bis*phosphatase (FBPase) is a key enzyme in gluconeogenesis. It is a potential drug target in the treatment of type II diabetes. The protein is also associated with a rare inherited metabolic disease and some cancer cells lack FBPase activity which promotes glycolysis facilitating the Warburg effect. Thus, there is interest in both inhibiting the enzyme (for diabetes treatment) and restoring its activity (in relevant cancers). The mammalian enzyme is tetrameric, competitively inhibited by Fructose 2,6-*bis*phosphate and negatively allosterically regulated by AMP. This allosteric regulation requires information transmission between the AMP binding site and the active site of the enzyme. A recent paper by Topaz et al. (*Bioscience Reports* (2019) **39**, pii:BSR20180960) has added additional detail to our understanding of this information transmission process. Two residues in the AMP binding site (Lys^112^ and Tyr^113^) were shown to be involved in initiating the message between the two sites. This tyrosine residue has recently be shown to be important with protein’s interaction with the antidiabetic drug metformin. A variant designed to increase metal ion affinity (M248D) resulted in a five-fold increase in enzymatic activity. Interestingly alterations of two residues at the subunit interfaces (Tyr^164^ and Met^177^) resulted in increased responsiveness to AMP. Overall, these findings may have implications in the design of novel FBPase inhibitors or activators.

## Fructose 1,6-*bis*phosphatase and disease

Fructose 1,6-*bis*phosphatase (fructose diphosphatase; FBPase; EC 3.3.1.11) catalyses the hydrolysis of fructose 1,6-*bis*phosphate to fructose 6-phosphate [1,2]. This reaction occurs in gluconeogenesis and in the Calvin cycle. In gluconeogenesis, it is one of three reactions which are not the exact reverse of the corresponding reaction in glycolysis. While ATP is required to phosphorylate fructose 6-phosphate in glycolysis, none is produced by the dephosphorylation in gluconeogenesis. The enzyme also serves as a key regulatory point, being inhibited by AMP and fructose 2,6-*bis*phosphate [3,4]. In contrast, the enzyme performing reverse reaction at this point in the glycolytic pathway, phosphofructokinase (PFK; EC 2.7.1.11) is activated by both AMP and fructose 2,6-*bis*phosphate [5–7]. The consequence of this is that, under conditions of low cellular ATP concentrations, FBPase is relatively inactive compared with PFK and ATP synthesis is stimulated. This avoids a ‘futile cycle’ in which fructose 1,6-*bis*phosphate is generated and hydrolysed, consuming ATP for no metabolic purpose [8].

FBPase is a homotetramer in most species studied to date, with yeasts being a notable exception [9,10]. Like many oligomeric enzymes it exhibits allosteric behaviour. Fructose 2,6-*bis*phosphate inhibition is competitive, with the compound binding to the active site and sterically hindering access by the substrate, fructose 1,6-*bis*phosphate [4]. In contrast, AMP binds at a separate site, distant from the active site. Its binding promotes a conformational change in the tetramer in which two subunits rotate by approximately 19° relative to the other two resulting in a less active form of the enzyme [11]. These two ligands do not act independently and there is known to be synergy between them: fructose-2,6-bisphosphate binding reduces the concentration of AMP required for a given level of inhibition [3,12]. It also induces positive co-operativity in the kinetics of the forward reaction, converting a hyperbolic (Michaelis–Menten) relationship between substrate concentration and rate into a sigmoidal one [3].

FBPase inhibition has been suggested as a potential therapy for type II diabetes [13,14]. In this disease, gluconeogenesis is a significant contributor to excess glucose. Reducing this excess would mitigate pathology related to high glucose concentrations in the blood and tissues. FBPase is an attractive target since its inhibition would only affect gluconeogenesis and not glycolysis. Furthermore, the existence of natural allosteric regulation of the enzyme suggests that it may be possible to mimic the effect of AMP, dramatically reducing its activity. Thus drug discovery efforts have focused on identifying molecules which recognise the AMP binding site, induce allosteric inhibition of FBPase but do not interact with other adenosine nucleotide binding enzymes. Some of these have demonstrated antidiabetic properties in cell and animal models [15–26]. Recently, the widely used type II diabetes drug, metformin, has been shown to act (at least in part) through the inhibition of FBPase, most likely by interaction at the AMP binding site [27].

FBPase is associated with a rare, autosomal recessive inherited metabolic disease (OMIM #229700). The incidence is estimated to be between 1/900000 and 1/350000 in European populations [28]. Disease-associated mutations include frameshifts, deletions, splice donor variants, and missense mutations. Relatively little work has been done on the consequences of the missense mutations on the enzymatic activity or stability of FBPase. Some variants (e.g. p.G164S, p.A177D and p.G261A) are inactive when expressed as recombinant proteins, suggesting that these point mutations result in significant changes to protein structure and/or folding [29,30]. Patients suffer from impaired gluconeogenesis and, consequently, hypoglycaemia, ketosis and lactic acidosis [31]. If left untreated, this can be fatal in newborn babies. However, if diagnosed early, interventions can be made which result in a good prognosis. These include avoidance of fasting, enrichment of the diet with glucose, and the reduction in fructose (and precursors such as sucrose) in the diet [32].

In clear cell renal cell carcinoma (ccRCC; the most common form of kidney cancer), cells lack FBPase activity, due to a chromosomal deletion [33]. Complementation of ccRCC cells with the wild-type FBPase gene inhibits their growth [33]. Thus lack of FBPase activity is necessary for the survival of these cells. Reduced FBPase activity has also been observed in some other forms of cancer cells (e.g. gastric, liver and cervical cancer) [34–36]. This effect is believed to be due to two factors. First, the loss of FBPase activity blocks gluconeogenesis and stimulates glycolysis [33]. Cancer cells typically rely heavily on glycolysis as a source of ATP and glycolytic intermediates, precursors of building blocks for synthetic reactions, even in aerobic conditions (the Warburg effect). Second, FBPase interacts with, and inhibits the activity of, hypoxia-inducible factors (HIFs). This inhibition promotes aerobic metabolism and restrains cell proliferation. Its loss results in a second factor which up-regulates the glycolytic pathway and removes a block on cell proliferation [33].

## Getting the message across

The existence of allostery in FBPase infers the presence of information transmission pathways within the protein. Typically, allosteric changes are transmitted through proteins by conformational changes, alterations in mobility or a combination of the two [37]. Crystal structures of FBPase with and without AMP bound have provided considerable information on the changes which occur at the ligand binding site and at the interface between subunits of the tetramer [11,38–40]. These structural studies have been complemented by site-directed mutagenesis work which has investigated hypotheses about the role of individual amino acid residues in mediating the conformational changes which accompany AMP binding [41–43]. Molecular dynamics simulations have been used to predict changes to mobility which occur as part of this conformational change. One consequence of these structural and dynamic changes is the displacement of a loop in the active site. This loop is critical for catalysis and its movement away from the substrate binding site renders the enzyme less active [44].

Recent work by Topaz et al. [45], published in Bioscience Reports extends our understanding of information transmission in mammalian FBPase. This study focused on seven variants: two which alter residues in a previously identified allosteric communication pathway and binding site for the allosteric inhibitor (4-(3-(6,7-diethoxy-quinazolin-4-ylamino)-phenyl)-thiazol-2-yl)-methanol (PFE; L56A and L73A); two affecting residues at the subunit interface (Y164A and M177A); two altering residues in the AMP binding site (K112A and Y113A) and one which changes a residue in a metal ion binding site (M248D).

As expected, alteration of the two lysine residues in the AMP binding site resulted in a lower responsiveness to regulation by this compound, but did not affect regulation by fructose 2,6-*bis*phosphate. The positively co-operative response to AMP was abolished by these changes. These variants also had increased catalytic efficiency (as measured by the specificity constant *k*_cat_/*K*_m_). A Y113F variant (Y114F in the numbering scheme used in the paper reporting this variant) has been recently reported to have greatly reduced sensitivity to AMP, but also to fructose 2,6-*bis*phosphate, suggesting that this is a critical region in the AMP binding site [27]. It appears that these residues are not just involved in the binding of AMP, but also in the initiation of information transmission to the active site.

Interestingly, the two variants located at the subunit interface resulted in a slight increase in responsiveness to both AMP and fructose 2,6-*bis*phosphate. Overall, they are less enzymatically active, but retain positive co-operativity towards AMP. The two variants in the allosteric communication pathway had little effect on AMP responsiveness or catalytic efficiency and a small effect on fructose 2,6-*bis*phosphate responsiveness. These are an interesting set of results since these changes might have been expected to disrupt communication. That they (largely) did not do so suggests the possibility of alternative information transmission pathways. Alternatively, it is possible that the alterations to these residues are not sufficient to fully disrupt the transmission of information. This may be particularly the case if protein mobility, as well as conformational changes, is important in mediating the allosteric effects. Such alterations are often critical in the transmission of information within proteins [37,46,47]. Further molecular dynamics simulations to understand the mobility of FBPase may yield a greater understanding of this aspect of information transmission.

While all the alterations of residues to alanine were designed to abolish interactions within the protein or between the protein and its ligands, the M248D variant was intended to increase the affinity for divalent metal ions. This variant has an impressive (greater than five-fold) increase in catalytic efficiency combined with a substantially reduced responsiveness to fructose 2,6-*bis*phosphate. As predicted, its affinity for magnesium is increased (six-fold, compared with the wild-type). Thus, the authors’ prediction that engineering FBPase to bind more tightly to the metal ion will result in increased activity was vindicated. Increases in catalytic efficiency and turnover are relatively rare outcomes of enzyme engineering experiments. In general, most changes reduce substrate affinities, turnover and efficiency [48]. The approach adopted here of increasing metal ion affinity is one which could, potentially, be adopted in other systems including those of biotechnological interest.

## Conclusion: implications for disease and treatment

While these experiments shed new light on the molecular mechanisms of information transmission in mammalian FBPase, the story is not complete. A map of the changes resulting from AMP binding and how these affect catalysis is not yet available. Nevertheless, each new set of data on FBPase variants which affect the process increases the knowledge base which can be exploited in the design of new inhibitors. The discovery that metformin targets this enzyme also strongly supports the proposition that new FBPase inhibitors may have potential as antidiabetic agents. Understanding these communication pathways between two separated sites (the AMP binding and the active site) in the regulation of the enzyme’s overall activity may also be important in determining the molecular changes which underpin FBPase deficiency. It is possible that some point mutations may affect this allosteric regulation, rather than the activity, of the enzyme. In cancers where lack of FBPase activity contributes to tumour progression, there may be a case for introducing the protein using strategies similar to those employed in enzyme replacement therapy for inherited metabolic diseases. Ideally, the protein used would be engineered for increased stability, activity and half-life. The M248D variant may be useful in this context. Finally, many enzymes exhibit allostery and insights gained in relatively well-characterised systems such as FBPase may well have applications in more poorly characterised or newly discovered enzymes.

## Abbreviations

ccRCC: clear cell renal cell carcinoma
FBPase: fructose 1,6-*bis*phosphatase
PFK: phosphofructokinase
